# Comparable cancer‐relevant mutation profiles in synchronous ductal carcinoma in situ and invasive breast cancer

**DOI:** 10.1002/cnr2.1248

**Published:** 2020-05-28

**Authors:** Helga Bergholtz, Surendra Kumar, Fredrik Wärnberg, Torben Lüders, Vessela Kristensen, Therese Sørlie

**Affiliations:** ^1^ Department of Cancer Genetics, Institute for Cancer Research Oslo University Hospital Oslo Norway; ^2^ Institute of Clinical Medicine, Faculty of Medicine University of Oslo Oslo Norway; ^3^ Department of Surgical Sciences Uppsala University Uppsala Sweden; ^4^ Department of Surgery Uppsala Academic Hospital Uppsala Sweden; ^5^ Department of Clinical Molecular Biology (EpiGen), Division of Medicine Akershus University Hospital Lørenskog Norway

**Keywords:** breast tumor progression, DCIS, invasive breast cancer, mutations, targeted sequencing

## Abstract

**Background:**

Ductal carcinoma in situ (DCIS) comprises a diverse group of preinvasive lesions in the breast and poses a considerable clinical challenge due to lack of markers of progression. Genomic alterations are to a large extent similar in DCIS and invasive carcinomas, although differences in copy number aberrations, gene expression patterns, and mutations exist. In mixed tumors with synchronous invasive breast cancer (IBC) and DCIS, it is still unclear to what extent invasive tumor cells are directly derived from the DCIS cells.

**Aim:**

Our aim was to compare cancer‐relevant mutation profiles of different cellular compartments in mixed DCIS/IBC and pure DCIS tumors.

**Methods and results:**

We performed targeted sequencing of 50 oncogenes in microdissected tissue from three different epithelial cell compartments (in situ, invasive, and normal adjacent epithelium) from 26 mixed breast carcinomas. In total, 44 tissue samples (19 invasive, 16 in situ, 9 normal) were subjected to sequencing using the Ion Torrent platform and the AmpliSeq Cancer Hotspot Panel v2. For comparison, 10 additional, pure DCIS lesions were sequenced.

Across all mixed samples, we detected 23 variants previously described in cancer. The most commonly affected genes were *TP53*, *PIK3CA*, and *ERBB2*. The *PIK3CA*:p.H1047R variant was found in nine samples from six patients. Most variants detected in invasive compartments were also found in the corresponding in situ cell compartment indicating a clonal relationship between the tumor stages. A lower frequency of variants were observed in pure DCIS lesions.

**Conclusion:**

Similar mutation profiles between in situ and invasive cell compartments indicate a similar origin of the two tumor stages in mixed breast tumors. The lower number of potential driver variants found in pure DCIS compared with the in situ cell compartments of mixed tumors may imply that pure DCIS is captured earlier in the path of progression to invasive disease.

**List of Abbreviations**
COSMIC: catalogue of somatic mutations in cancer
DCIS: ductal carcinoma in situ
ER: estrogen receptor
IBC: invasive breast cancer
LCM: laser capture microdissection
PALM: polyethylene membrane
PCR: polymerase chain reaction
PGM: personal genome machine
PR: progesterone receptor
qPCR: quantitative polymerase chain reaction

## 1 INTRODUCTION

Ductal carcinoma in situ (DCIS) is a noninvasive breast cancer. In DCIS, abnormal cells are contained within the milk ducts while the basement membrane is intact, and there is no invasion of surrounding stroma.^1^ Today, DCIS comprises about 20% of all breast carcinoma diagnoses, usually detected in the context of mammography screening.^2^ In situ lesions are generally accepted as nonobligate precursors to invasive breast cancer (IBC), but importantly, not all in situ lesions progress to become invasive. There is however, an increased risk of developing IBC subsequent to an in situ carcinoma if left untreated.^3^, ^4^ The clinical challenge is therefore to distinguish high risk from low risk lesions in order to offer optimal treatment to these patients.^5^ Much remains to be learned about the pathogenesis of DCIS to be able to predict disease progression of these nonobligate breast cancer precursors.

Many cases of breast cancer present as mixed lesions, that is, synchronous invasive ductal carcinoma and DCIS. In such cases, the in situ lesion is thought to be the precursor of the invasive tumor and studies have reported an overall high degree of similarity of genetic aberrations between DCIS and IBC.^6^, ^7^, ^8^, ^9^, ^10^ Nevertheless, differences in type and frequency of mutations have also been reported.^11^ It has been hypothesized that DCIS and IBC originate from the same ancestor cell, but have deviated prior to the in situ stage following separate tumor progression paths.^12^ In tumors without an in situ compartment the invasive carcinoma may have arisen de novo,^13^ or the preinvasive stage has been a brief, transient phase along the progression to invasive breast carcinoma.^14^ More, in‐depth sequencing studies are required to investigate the intralesion heterogeneity in DCIS and whether progression to IBC is a result of clonal selection.^6^, ^15^

In this study, we have sequenced microdissected cell compartments from 26 mixed breast tumors using the Ion AmpliSeq Cancer Hotspot Panel v2. The mutation spectrum across 44 samples of carcinoma in situ, invasive carcinoma, and adjacent normal tissue showed a high degree of similarity between synchronous DCIS and IBC and a higher mutation frequency in the in situ cell compartment in mixed tumors compared with pure DCIS.

## 2 MATERIALS AND METHODS

### 2.1 Tumor tissue samples

Fresh frozen tissue from patients with mixed tumors (invasive ductal carcinoma with synchronous in situ lesion) or pure DCIS was collected at the Fresh Tissue Biobank, Department of Pathology, Uppsala University Hospital, Sweden. Histopathological evaluation of all cases was performed by a pathologist. DCIS tumor compartments were given a histopathological grade using the EORTC system^16^ while invasive compartments were graded using the Elston & Ellis system.^17^ Estrogen receptor (ER) and progesterone receptor (PR) status was determined by immunohistochemistry, and were previously published.^18^ Samples were considered ER/PR positive if >10% of the cells showed positive nuclear staining. HER2‐status was determined using Silver‐enhanced in situ hybridization and scored as previously described.^18^

### 2.2 Laser capture microdissection

Invasive, in situ and normal cell areas were microdissected using laser capture microdissection on a Zeiss inverted microscope PALM Laser MicroBeam System (Carl Zeiss, Germany) as previously described.^8^ Frozen 14 μm‐thick sections were mounted on polyethylene membrane (PALM) covered slides and stained with hematoxylin (60 μL) mixed with RNasin for 1 minute, incubated in Zincfix (60 μL) for 30 seconds, followed by a series of 30‐seconds incubation steps in 75%, 95%, and 100% ethanol, respectively. Adjacent 4 μm‐thick sections were cut and stained by a routine hematoxilin and eosin protocol to locate the areas to be microdissected. Cells were captured into collecting caps and preserved in 50 μL Trizol at −80°C for DNA extraction. The number of cells obtained was estimated by the operator during microdissection and between 100 and 4000 cells were obtained for each sample. Pure DCIS samples were not microdissected; for these samples, whole FFPE tumor sections were used for DNA isolation.

### 2.3 DNA purification

DNA was isolated using Qiagen (Hilden, Germany) DNeasy Blood and Tissue Mini Kit. Samples were thawed and centrifuged at 16 000*g* for 15 minutes to precipitate DNA. After complete removal of Trizol, 180 μL buffer ATL and 20 μL protease was added and the tubes incubated at 56°C overnight before addition of 200 μL buffer AL. Samples were mixed well by vortexing before 200 μL ethanol was added and the samples were again mixed well by vortexing. The samples were then transferred to DNeasy Mini spin columns and further processed as per the manufacturer's instructions before DNA was eluted in 100 μL buffer AE. To improve recovery of the DNA, the elution buffer was left on the columns for 5 minutes before a final centrifugation step. For quantification and quality assessment of the DNA, quantitative polymerase chain reaction (qPCR) was performed with the KAPA hgDNA Quantification and QC Kit (KAPA Biosystems, Wilmington, MA) as per the manufacturer's instructions. Isolation of DNA from pure DCIS tumors were performed using the QIAcube system with the AllPrep DNA/RNA Universal Kit (cat.no. 80224, Qiagen, Hilden, Germany) according to protocol provided by the supplier.

### 2.4 Library preparation

Sequencing libraries for Ion Torrent sequencing were prepared using the Ion Torrent AmpliSeq Library Kit 2.0 (Thermo Fisher Scientific, Waltham, MA), and the Ion AmpliSeq Cancer Hotspot Panel v2 and Sample ID Panel as per the manufacturer's instructions. Briefly, approx. 100 pg DNA was mixed with Ion AmpliSeq HiFi Master Mix and the two primer pools, and amplified for 27 cycles followed by partial digestion of the primer sequences and ligation of barcoded adapters. The libraries were purified using Agencourt AMPure XP beads (Beckman Coulter, Brea, CA) and amplified by polymerase chain reaction (PCR) for 5 cycles followed by a two‐round purification process with AMPure XP beads. The final libraries were quantified on Agilent Bioanalyzer instrument (Agilent Technologies, Santa Clara, CA) with the Agilent High Sensitivity DNA Kit and stored at −20°C. The AmpliSeq Cancer Hotspot Panel yields 207 amplicons that cover hotspot regions of 50 relevant cancer genes (Suppl. file 1).

### 2.5 Template preparation and sequencing

Libraries were normalized to 100 pM in Low TE and equal amounts of each library were pooled. Each pool was diluted 10 times and 20 μL were clonally amplified on the Ion OneTouch system using the Ion OneTouch 200 Template Kit v2 DL and enriched with the Ion OneTouch ES as per the manufacturer's instructions. Sequencing was carried out on the Ion Torrent Personal Genome Machine (PGM) using the Ion PGM 200 Sequencing Kit and Ion 314 or Ion 316 Chips for 400 cycles according to the manufacturer's instructions. For the microdissected samples, mean number of mapped reads was 395 584 (range 126 272‐933 608), mean read length 108 bp (range 73‐115 bp) while mean depth was 1655 (range 411‐3922). For the pure DCIS samples mean number of mapped reads was 273 769 (range 227 290‐345 207), mean read length 113 (range 112‐116 bp), and mean depth 1262 (range 1046‐1585).

### 2.6 Variant calling

Data was analyzed using the AmpliSeq Variant Caller plug‐in within the Ion Torrent Suite software (version 5.0.4, Thermo Fisher Scientific) with Human genome assembly build 37 (GRCh37) as reference. In total, 57 samples were sequenced. Three samples were excluded from further analysis after quality assessment and altogether were 44 microdissected samples from 26 mixed tumors and 10 pure DCIS successfully sequenced. Due to low input and varying sample quality for the microdissected samples, a strict cut‐off was applied; only variants with maximum allele frequency >10% and quality >100 across all microdissected samples were included. For the pure DCIS samples, variants with allele frequency <10% were also included since these data were of high quality. Variants were manually assessed in Integrative Genomics Viewer^19^ to evaluate strand bias and potential technical artifacts. Variants were filtered against the Genome Aggregation Database version 2.1.1^20^ to exclude single nucleotide polymorphisms.

### 2.7 Digital droplet PCR

Digital droplet PCR was performed using the RainDrop system (RainDance technologies) to validate the *PIK3CA*:H1047R variant found in nine samples on the Ion Torrent platform. DNA was isolated from separate FFPE tumor sections using DNeasy Mini spin columns as described above. An assay with two color fluorescent TaqMan probes was used to discriminate between droplets containing mutant and wild type alleles. A 50 μL reaction mix containing 2x KAPA Probe Force qPCR Master Mix (Sigma Aldrich, St. Louis, MO), 25x Droplet Stabilizer (RainDance Technologies), 13.3 μL nuclease free H_2_O, 9 μL DNA sample, and 0.7 μL primer/probe mix with 500 nM fwd/rev primer and 200 nM WT/mutant probe was made for each sample. The total reaction mix was loaded onto the RainDance Source chip for partitioning of the mix into millions of single droplets. Each droplet contains a PCR mix‐oil emulsion and a single DNA fragment (positive) or no target molecule (negative). After partitioning, a PCR amplification was performed, where each droplet acts as an individual PCR reaction. The PCR conditions were as follows: 98°C (3 minutes), 55 cycles of 95°C (10 seconds), and 60°C (1 minute) with ramp speed of 0.5°C/second, 72°C (10 minutes), 98°C (10 minutes), 12°C (10 minutes), and keep at 12°C. The samples were transferred to the RainDrop Sense instrument for automatic counting of positive and negative droplets depending on the presence or absence of a fluorescent signal enabling calculation of the absolute number of targets present in the original sample.

### 2.8 Statistical analysis

Fisher's exact tests were performed to test whether there was any statistically significant association between variants in genes and ER or PR status.

## 3 RESULTS AND DISCUSSION

In total, 19 invasive carcinoma, 16 DCIS and nine normal, microdissected tissue samples from 26 patients with mixed tumors, were subjected to targeted sequencing of 50 oncogenes and tumor suppressor genes. Amongst the samples were 13 in situ/invasive pairs and from three of these, adjacent normal epithelial cells were also sequenced. In addition, we sequenced 10 pure DCIS samples. An overview of relevant clinical information is shown in Suppl. file 2. Mean age at time of diagnosis was 52 years (range 30‐81) and mean tumor size was 23.7 mm (range 1‐80). Of all tumors, 28/36 (78%) were ER positive and 23/36 (64%) PR positive. All PR‐positive tumors were ER‐positive.

**FIGURE 1:**
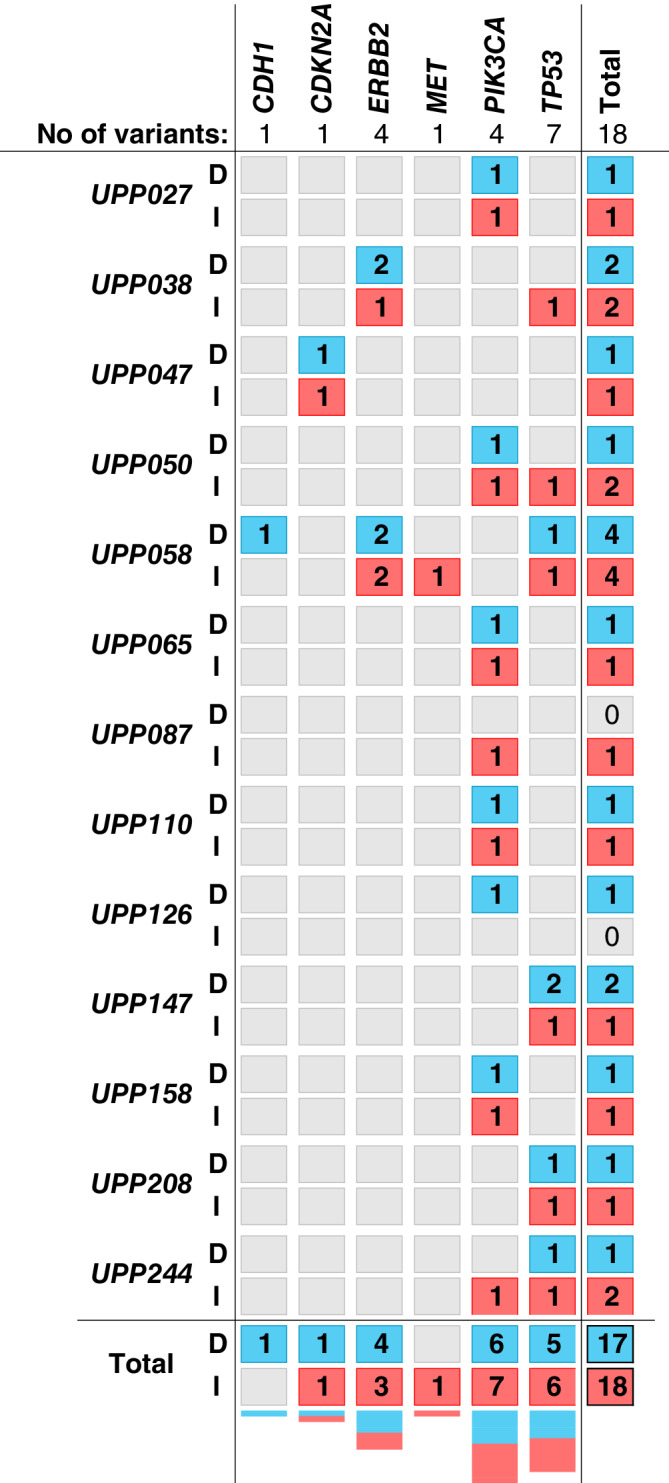
Genes with pathogenic variants identified in the 13 available DCIS/IBC sample pairs. DCIS (blue), IBC (red). DCIS, ductal carcinoma in situ; IBC, invasive breast cancer

Across all samples from mixed tumors we identified 23 different, potentially pathogenic variants in seven genes (*AKT1, CDH1, CDKN2A, ERBB2, MET, PIK3CA,* and *TP53*) (Suppl. file 3). *PIK3CA* and *TP53* were the most commonly mutated genes. Most variants were present in only one patient; but for two of the genes (*AKT1* and *PIK3CA*), identical variants were identified in more than one patient. The number of variants in each in situ or invasive cell compartment ranged from zero to four, and most tumors (12/16 in situ, 13/19 invasive) carried only one variant. The most common variant (*PIK3CA*:p.H1047R) was found in nine samples from six patients. Across the samples from mixed tumors, five different *PIK3CA* variants were detected in 19 samples from 13 patients including one normal sample. By contrast, none of the nine *TP53* variants were found in more than one patient. Among the 13 cases of pairs, for which both in situ and invasive samples were available from the same patient, we found 18 variants in six different genes (Figure 1). In six of the cases, the variant(s) were identical in both compartments.

Interestingly, we found five different *ERBB2* variants; two (p.D769H and p.V777L) resided in both the in situ and invasive tumor compartments of the same tumor. Two other *ERBB2* variants were found in a second tumor. One of these (p.D769Y) was found in both the in situ and invasive tumor compartments and the other (p.L755S) was found only in the in situ compartment. One *ERBB2* variant (p.G776delinsAVGC) was found in a normal sample; however, none of the corresponding tumor cell compartments were successfully sequenced for this particular case. Altogether, these findings demonstrate large intertumor heterogeneity in mutation pattern in synchronous DCIS and IBC and indicate that *ERBB2* variants also are present early in tumorigenesis.

**FIGURE 2:**
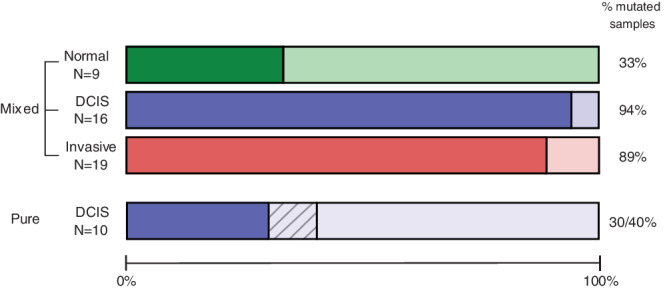
Frequency of samples with pathogenic variants. The bar plot illustrates the frequency of samples with (dark color) and without (light color) any pathogenic variant (frequency indicated at the right). The hatched area in the pure DCIS bar illustrates the frequency of variants with allele frequency below 10%. DCIS, ductal carcinoma in situ

In three tumors (UPP027, UPP208, and UPP244), normal epithelium was successfully sequenced in addition to invasive and in situ tumor tissue. Two of these tumors (UPP027 and UPP208) carried only one variant each (*PIK3CA*:p.H1047R and *TP53*:p.A84fs, respectively) and none of the corresponding normal compartments carried these variants. The last tumor with three samples (UPP244) carried two different variants; one of these (*TP53*:p.R175H) was found in both the in situ and invasive compartments, while the other (*PIK3CA*:p.H1047R) was found in the normal compartment at a frequency of 30%, absent in the in situ compartment and present at a very low frequency (3%) in the invasive compartment. Across the cohort, nine normal breast epithelium samples were sequenced, and amongst these, three carried potentially pathogenic variants (Figure 2). However, since all normal tissue samples sequenced in this study were obtained adjacent to tumor areas, they should not be considered entirely normal.

We found a significant association between *PIK3CA* variants and positive PR status (*P* = .039, Fisher's exact test), which has been previously noted.^21^, ^22^, ^23^ A similar association was not seen for ER (*P* = .44), however; the low number of samples in this study may have prevented the identification of any such association.

In addition to microdissected tissue from mixed tumors, we sequenced 10 pure (nonmicrodissected) DCIS. Three variants in three different tumors were detected; *PIK3CA*:p.C420R, *PIK3CA*:p.E542K, and *TP53:*p.R213X (Suppl. file 3). Two of these were not detected in any of the microdissected samples, while the third variant (*PIK3CA*:p.C420R) was found in one of the invasive tumor cell compartments, but was filtered out due to allele frequency below threshold. There was a notable difference in the number of variants across the 50 genes between in situ cell compartments from mixed tumors compared with pure DCIS. Almost all in situ cell compartments from the mixed tumors, 15/16 (94%), carried at least one variant while only 3 out of 10 (30%) of the pure DCIS tumors carried any of the variants (Figure 2). However, the pure DCIS tumors were not subjected to microdissection before sequencing which may have lowered the allele frequency and compromised the detection of relevant variants. When including all variants regardless of allele frequency, three additional variants were found (Suppl. file 3). Using this cut‐off, 4/10 (40%) of the pure DCIS carry potentially pathogenic variants (Figure 2), however; comparison with the sequencing results from synchronous DCIS from mixed tumors is challenging when different thresholds are applied. Noticeably, none of the pure DCIS tumors carried the most common variant identified among the mixed tumors (*PIK3CA*:p.H1047R). Targeted sequencing as performed here includes only a limited number of genes and therefore we cannot exclude the possibility that mutation spectra across other putative driver genes might be similar between the two different types of in situ cancers. It also important to recognize that the pure DCIS tumors in this cohort may be earlier in the progression path to invasiveness and therefore carry fewer tumor driving mutations. Finally, heterogeneity at the DCIS stage may imply that some pure DCIS are on the verge of becoming invasive and display a mutational pattern similar to IBC whereas others will never become invasive and that these could be regarded as different entities. These findings confirm those of other studies^24^, ^25^ and highlight the importance of being conscious about distinguishing synchronous DCIS from pure DCIS lesions when studying tumor progression.

When DCIS presents synchronous with invasive disease, it is unclear whether these multiple stage‐specific cell populations have a common ancestor or develop from multiple clones. Previous sequencing studies have reported similar mutation profiles in DCIS and IBC, with *PIK3CA*, *TP53*, and *GATA3* as the most commonly affected genes.^8^, ^9^, ^10^, ^24^, ^26^, ^27^, ^28^, ^29^, ^30^ However, different prevalence of *PIK3CA* variants has been observed between DCIS and IBC. One study reported *PIK3CA* variants restricted to the in situ compartment in two cases of synchronous DCIS and IBC, while in a third case, a reduced frequency of a specific *PIK3CA* variant was found in invasive cells relative to the cells from the in situ compartment.^9^ In one tumor in our study, we found a *PIK3CA* variant in the in situ cells, and not in the invasive cell compartment, while in two tumors, we found a *PIK3CA* variant in the invasive cells while not in the corresponding in situ cell compartment. In our study, the sequencing panel did not include *GATA3*, so the high frequency of *GATA3* variants previously found in DCIS could not be confirmed.^27^

Ion semiconductor sequencing is a “sequencing by synthesis” method based upon detection of hydrogen ions that are released during polymerization of DNA. The technology is well suited for targeted sequencing of samples with minute amounts of DNA which is often the challenge with microdissected tissue. This has allowed us to sequence a panel of the most frequently mutated genes in cancer, in relatively few cells from stored Trizol cell fractions after microdissection. To validate our findings, we used digital droplet PCR to quantify the most frequently detected variant in this study, *PIK3CA*:p.H1047R and found similar frequencies as by sequencing (Table 1). Four of the samples with *TP53* mutations in this study were included in a previous study of *TP53* mutations in synchronous DCIS and IBC.^8^ In three of these samples (UPP038, UPP208, UPP142 [pure DCIS]), we detected the same variants, while for UPP065, a 10 bp deletion of codons 106‐109, was not called by the Ion Torrent analysis pipeline. However, we identified the deletion in our data by manual inspection. This discrepancy could be due to inaccurate flow‐calls, a known artifact of PGM, which may cause homopolymers to be under‐called.^31^

**TABLE 1:** Validation results ddPCR

SampleID	Input (ng DNA)	WT droplets	MUT droplets	Mutation frequency ddPCR %	Mutation frequency Ion Torrent %	Comments
UPP027 Normal	0.054	66	0	0	0	
UPP027 DCIS	0.297	108	50	32	33	
UPP027 Invasive	0.01	58	20	26	30	
UPP050 Normal	NA	10 187	2	0.02	NA	Ion Torrent sequencing failed
UPP050 DCIS	0.021	2639	1279	33	43	
UPP050 Invasive	0.058	7330	20	0.27	0	
UPP087 Normal	NA	10 957	567	5	NA	Ion Torrent sequencing failed
UPP087 DCIS	0.016	0	0	NA	0	ddPCR failed
UPP087 Invasive	0.062	1102	272	20	40	
UPP158 DCIS	0.085	169	88	34	41	
UPP158 Invasive	0.156	288	184	39	18	
UPP233 Invasive	0.144	14	6	30	27	
UPP244 Normal	0.063	1	0	NA	29	ddPCR failed
UPP244 DCIS	0.144	2115	0	0	0	
UPP244 Invasive	0.081	43 281	1	0.002	3	
*WT‐PIK3CA_ctr*	*10*	*7596*	*0*	*0*	*Not applicable*	*Negative control*
*PIK3CA_5_ctr*	*50*	*40 733*	*3091*	*7*	*Not applicable*	*Positive control*
*PIK3CA_5_ctr*	*50*	*43 064*	*3139*	*7*	*Not applicable*	*Positive control*

*Note*: PIK3CA:p.H1047R.

## 4 CONCLUSION

In this study, we performed targeted sequencing of microdissected tissue from in situ and invasive tumor cell compartments from 26 patients with mixed DCIS/IBC tumors, in addition to 10 pure DCIS tumors. Although the number of cases included was small, we found that across the 50 cancer‐relevant genes included in the panel, the spectrum of variants was similar between synchronous DCIS and IBC supporting earlier findings of a clonal relationship between the two tumor stages and a possible selection of subclones during tumor progression. *PIK3CA* and *TP53* were the most frequently mutated genes and alterations occurred at the DCIS stage or possibly earlier. Sequencing of 10 pure DCIS indicated a lower number of potentially pathogenic variants in these lesions compared with synchronous DCIS.

## CONFLICT OF INTEREST

The authors have stated explicitly that there are no conflicts of interest in connection with this article.

## AUTHORS' CONTRIBUTIONS

All authors take responsibility for the integrity of the data and all authors approved the final manuscript. *Conceptualization*, V.K.,T.S.; *Methodology*, H.B., S.K.; *Investigation*, H.B., S.K.; *Formal Analysis*, H.B. and S.K.; *Resources*, F.W., V.K., T.S.; *Writing ‐ Original Draft*, H.B., T.S.; *Writing ‐ Review & Editing*, H.B.,F.W., V.K., T.S.; *Visualization*, H.B.; *Supervision*, V.K., T.S.; *Funding Acquisition*, V.K., T.S.

## DATA AVAILABILITY STATEMENT

The data that support the findings of this study are available from the corresponding author upon reasonable request.

## ETHICS STATEMENT

The study complies with the Declaration of Helsinki, and was approved by the Ethics Committee at Uppsala University Hospital, Sweden (approval number 2005/118). In this retrospective cohort study informed consent from the women was not needed according to the ethical approval.

## Supporting information


**Suppl. file 1** Overview of the 50 genes included in the AmpliSeq Cancer Hotspot Panel (.xlsx)

**Suppl. file 2** Clinical information and overview of samples analyzed (.xlsx)

**Suppl. file 3** List of all pathogenic variants detected (.xlsx)
